# A comparative screening of laccase-mediator systems by white-rot fungi laccases for biocatalytic benzyl alcohol oxidation

**DOI:** 10.1038/s41598-022-24839-6

**Published:** 2022-12-14

**Authors:** Ivana Marino, Eugenia Pignataro, Donatella Danzi, Francesco Cellini, Cosimo Cardellicchio, Antonino Biundo, Isabella Pisano, Maria Annunziata M. Capozzi

**Affiliations:** 1 Agenzia Lucana di Sviluppo e Innovazione in Agricoltura (ALSIA), Centro Ricerche Metapontum Agrobios, SS. Jonica 106, Km 448, 2, 75012 Bernalda, Italy; 2 Department of Chemistry, University of Bari Aldo Moro, Via Edoardo Orabona 4, 70125 Bari, Italy; 3 CNR ICCOM, Department of Chemistry, University of Bari Aldo Moro, Via Edoardo Orabona 4, 70125 Bari, Italy; 4 Department of Biosciences, Biotechnology and Biopharmaceutics, University of Bari Aldo Moro, Via Edoardo Orabona 4, 70125 Bari, Italy; 5 Institute of Biosciences and Bioresources, National Research Council, 70126 Bari, Italy

**Keywords:** Biotechnology, Green chemistry, Fungi

## Abstract

Production of value-added compounds from waste materials is of utmost importance for the development of a sustainable society especially regarding their use as catalysts in industrially relevant synthetic reactions. Herein, we show the production of laccases from four white-rot fungi, which were grown on agricultural residues, specifically *Trametes versicolor *11269, *Pleurotus ostreatus* 1020, *Panus tigrinus* 707 and *Lentinula edodes* SC-495. The produced laccases were tested on a laccase-mediator system (LMS) for the biocatalytic oxidation of the model substrate benzyl alcohol into benzaldehyde. The LMS was carried out in the presence both of tetrahydrofuran as co-solvent and of the mediator 2,2,6,6-tetramethyl-1-piperidinyloxyl (TEMPO) due to its high redox potential and its ability to perform the oxidation. Tolerance studies showed that the dialyzed solutions were able to tolerate 1% (99:1 v/v) of co-solvent, whereas a concentration of 10% *v/v* had a detrimental activity. Performances in the biocatalytic oxidation of laccase solutions from different purification steps were compared. Similar conversion was observed for laccase in dialysis (raw) and gel filtration (GF) product versus commercial *T. versicolor* laccase. The latter oxidized almost 99% of substrate while the other laccase solutions were able to reach a conversion from 91% for the laccase solution from *P. tigrinus* 707 after dialysis, to 50% for the laccase solution from *P. ostreatus* 1020 after gel filtration. This work highlights the potential of unpurified laccase solutions to be used as catalysts in synthetic reactions.

## Introduction

The Green Transition is pushing towards the use of renewable resources to produce heat and energy, for a better employment of natural resources, such as solar, wind and hydropower energy. To produce chemicals, biomasses are used as sustainable alternatives to fossil resources by biorefining, which is one of the main drivers of bio-based economy^1^ . The use of agro-industrial waste has been reported in solid-state fermentation (SSF) due to the accessibility of biomass^2^. SSF has many advantages, such as higher product yield, lower wastewater output, simpler fermentation media and reduced energy requirements^3^.

Industrially relevant enzymes can be produced by solid-state fermentation for their deployment in organic synthesis. In this context, the laccases have been received enormous attention. Laccases (EC 1.10.3.2, benzenediol:oxygen oxidoreductases) are a well explored group of enzymes belonging to the multi-copper oxidase (MCO) family. Although laccases are widespread in nature, as they have been described from bacteria, fungi, higher plants and insects, fungal laccases represent the most significant group of the “blue” MCO family regarding the number and characterizations. Typical fungal laccases are 60–70 kDa monomeric glycoproteins with a well characterized catalytic mechanism for the formation of radical species^4^. They contain four copper ions which are organized in two sites. The type 1 copper receives one electron from the substrate and transfers it via a His-Cys-His motive to the trinuclear center. The trinuclear center contains one type 2 and two type 3 copper ions and this is the site where the oxygen reduces to water. In this reaction, four electrons from substrate molecules are transferred to one molecule of O_2_, reducing it to two molecules of water^5^. Plant laccases play an important role in the synthesis of lignin, while fungal laccases catalyze its degradation for wood-decay, pathogenesis, fungal morphology and detoxification^6^.

These natural activities are exploited in several applications by different industrial processes^7,8^ . Unlike other enzymes, laccases can oxidize various substrates, such as phenols^9^, aromatic and aliphatic amines and some inorganic ions, while reducing oxygen to water^10^ . In the food and textile industry, laccases are used to reduce the oxygen content, specifically in beer production to increase the product shelf-life and for their bleaching activities on denim fabrics, respectively^11^ . Moreover, different studies showed the ability of laccases to produce polymeric structures^12–14^ .

The catalytic cycle of laccases (redox potential 0.5–0.8 mV vs. normal hydrogen electrode) can only start if the substrate of interest has the appropriate redox potential^15^ . Through “Laccase-mediator system” (LMS), radicalic species may play a role as mediators in non-phenolic compounds oxidation. Indeed, due to steric hindrance and/or redox potential incompatibility of substrates, mediators can act as redox intermediates, following a nature mimicking strategy.

**Figure 1:**
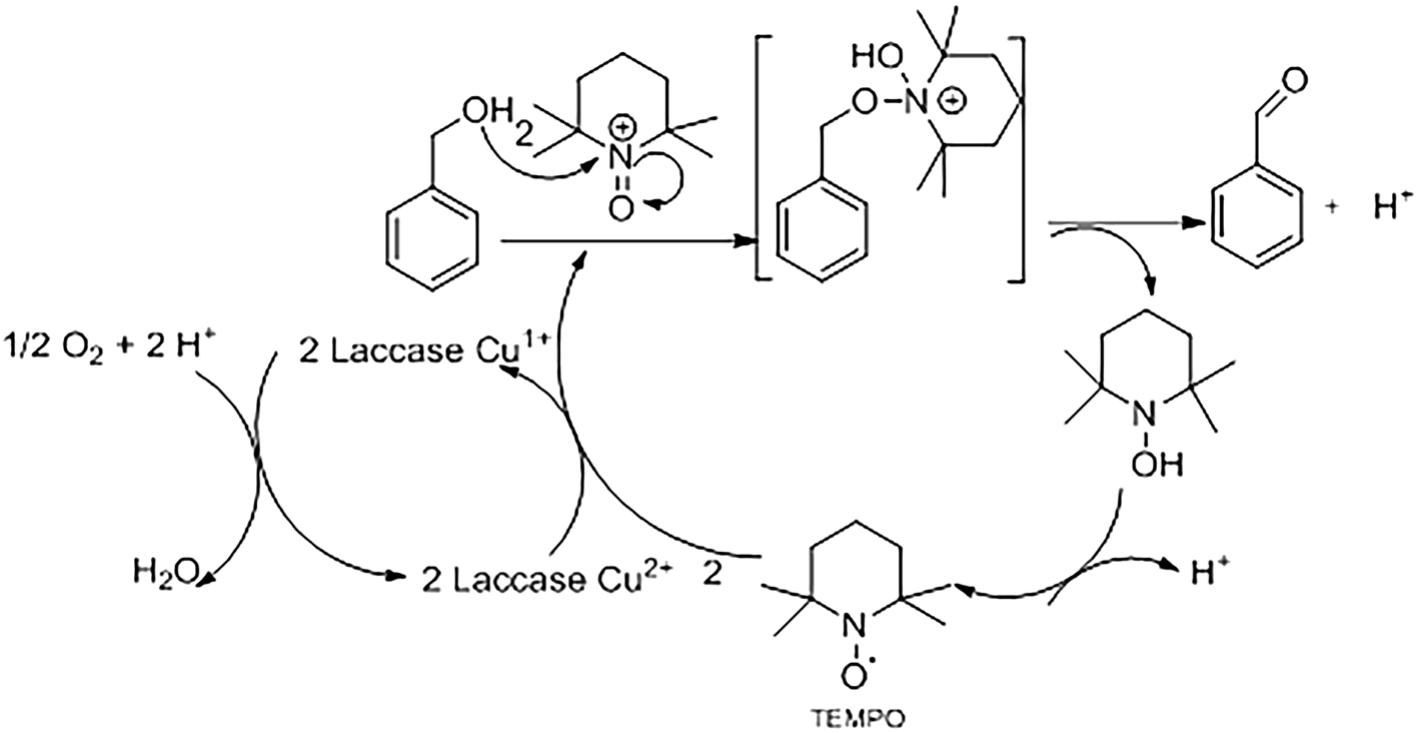
Reaction mechanism of benzyl alcohol oxidation by oxygen catalyzed by laccase/TEMPO.

To be considered a proper mediator, the compound must not inhibit the enzyme and its conversion must be cyclic. One of the most common mediators in LMSs is the compound 2,2,6,6-tetramethyl-1-piperidinyloxyl (TEMPO), which has sufficiently high redox potential and it is more efficient than other mediators. The TEMPO is present in the solution in the form of a relatively stable N-oxyl radicalic which is also able to oxidize (Fig. 1) non-phenolic structures^16,17^ .

Shortly, laccases oxidize TEMPO to produce the oxo-ammonium ion, which reacts with the substrate^18^ . Proton removal generates the oxidized product and the reduced (N–OH) form of TEMPO. The reduced TEMPO is converted by laccases to the oxidized form and then to the oxo-ammonium ion^19,20^. The LMS-TEMPO, is one of the most studied systems for converting primary (Fig. 1) and secondary alcohols into oxidized compounds, such as aldehydes, acids and ketones. The selective oxidation of alcohols to the corresponding carbonyl compounds is of utmost importance in both academia and industry. Primary alcohols are directly oxidized to carboxylic acids by H_2_CrO_4_ or KMnO_4_. A selective oxidation to aldehyde, instead, can be achieved using pyridinium chlorochromate (PCC) in stoichiometric amounts in aprotic solvents such as dichloromethane. Catalytic oxidation, instead, requires metal catalysts which are expensive and toxic^21^. An eco-sustainable alternative is the use of Laccase/TEMPO-mediator systems, that has been used for the biocatalytic selective conversion of alcohols to aldehydes^22^. The application of enzymes in synthetic chemistry requires the use of non-conventional reaction systems to dissolve hydrophobic substrates in the presence of water, including the use of organic solvents ^4,23^. Both water-miscible solvents^24^ and biphasic water-immiscible solvents^25,26^ have been applied to improve substrate solubility.

In the present work, we compare enzymatic activity of crude and purified laccase-containing mixtures produced from four edible mushrooms which were grown through SSF using wheat straw as substrate; the model reaction is the oxidation of benzyl alcohol to benzaldehyde through the laccase/TEMPO LMS system.

## Materials and methods

### Chemicals and reagents

All chemicals and reagents used in this work were of analytical grade. Potato Dextrose broth (PDB), ammonium sulfate, Tris–HCl, benzyl alcohol, 2,2,6,6-tetramethyl-1-piperidinyloxyl (TEMPO), 2,6-dimethoxyphenol (2,6-DMP) were purchased from Sigma-Aldrich (USA). Commercial laccase from *Trametes versicolor* (TV σ) was purchased from Sigma-Aldrich (USA). Organic solvents were purchased at the higher commercial quality and used without further purification.

### Strain and culture media

Four white-rot fungi were used in this work: *Lentinula edodes* SC-495 and *Panus trigrinus* 707, belonging to the culture collection of ALSIA; *Pleurotus ostreatus* 1020 and *Trametes versicolor* 11269, were purchased from the strain collection of the DSMZ (Deutsche Sammlung von Mikroorganismen und Zellkulturen). These fungal cultures were cultivated in slant tubes using PDB as medium containing 25 g L^−1^ agar at 28 °C for 7 days. For each strain, a liquid pre-inoculum was prepared by adding 30 mL sterile water into the slant tube to resuspend the mycelium. To disrupt the mycelium, the suspension was then homogenized at 24,000 rpm for 30 min by a T25 Ultra-Turrax (IKA, Germany). An aliquot of 25 mL was then transferred into a sterile Erlenmeyer flask with 100 mL fresh PDB medium. The strains were grown at 28 °C for 7 days at 180 rpm. After further homogenization, 25 mL were transferred into a 250 mL fresh PDB medium and grown at 28 °C for 5 days at 180 rpm.

### Agricultural biomass

Dry Saragolla (*Triticum turgidum* subsp. *durum*) straw was collected from Metapontum Agrobios Research Center (Italy) fields. The Saragolla grain is an early durum wheat variety with an exceptionally high and stable production potential in terms of biomass and grain yield^27,28^. Wheat bran was used as co-substrate for the mycelia growth and for laccase production with a composition as previously reported^29^ . All the experimental work on plant material described in this study complies with the relevant institutional, national, and international guidelines and legislation.

### Solid-state fermentation for laccase production

Grinded straw/bran mix (100 g), with a straw:bran ratio of 4:1 *w/w*, containing 70% moisture content (233 mL 0.5 mM Cu_2_SO_4_ solution), were inserted in an autoclavable plastic bag and sterilized by autoclave at 121 °C ^29,30^. After cooling the mixture at room temperature, 30 mL liquid inoculum from a 250 mL culture, containing a concentration of 3 mg mL^−1^ (dry weight of the mycelium) from each strain, were added and properly mixed to accurately spread fungal cells. The bags were kept in the dark at 28 °C for 7 days.

### Extraction and purification of laccase

To extract the extracellular liquor containing enzymes and organic compounds from the SSF batch, 100 g of biomass was pressed through a hydraulic press (Ravaglioli Spa, Italy) with a maximum pressure of 415 bar and a maximum capacity of 15,000 kg. To remove solid particles, a volume of 150 mL extracted liquor was centrifuged at 15,000 rpm for 25 min at 4 °C.

The prepared supernatant was then mixed with (NH_4_)_2_SO_4_ to reach 70% *w/v* saturation. The mixture was stored at 4 °C under magnetic stirring for 24 h. To collect precipitated proteins, the mixture was then centrifuged at 10,000 rpm for 30 min at 4 °C. The protein pellet was then solubilized in 0.05 M Tris–HCl pH 7.8 and centrifuged at 4,000 rpm for 10 min at 4 °C to remove impurities. To remove the excess of (NH_4_)_2_SO_4_ from the solution, dialysis was performed into a dialysis tube of 14 kDa cut-off and dialyzed against 2 L 0.05 M Tris–HCl pH 7.8 overnight at 4 °C under magnetic stirring.

To further purify the liquor of each strain, 30 mL protein solution were loaded onto an ion-exchange chromatography column packed with Sepharose Q as stationary phase connected to a Fast Protein Liquid Chromatography (FPLC) ÄKTA system (GE Healthcare, Sweden). The column was equilibrated with solution A, which contained 50 mM Tris–HCl, 15 mM NaCl pH 7. The elution was allowed using solution B, which contained 50 mM Tris–HCl, 1 M NaCl pH 7.8 with a flow of 1 mL min^-1^ and a gradient from 0 to 100% solution B within 50 min. The protein elution was detected with a UV detector at 280 nm.

For each strain, fractions rich in laccase activity were combined and concentrated for the next purification step of gel filtration chromatography. A 60-cm column was packed with Toyopearl resin 50H (TosoH Bioscience, Japan) and connected to an FPLC ÄKTA system. Proteins were eluted with 50 mM Tris–HCl pH 7.8. Fractions which contained laccase activities were collected and stored at −20 °C until further analysis.

### Laccase activity assay

Enzyme solutions were spectrophotometrically assayed at 477 nm using 2,6-dimethoxyphenol (2,6-DMP) as substrate at 30 °C (ε_477_ = 14,600 M^−1^ cm^−1^) ^31^ . The assay mixture consisted of 2 mM 2,6-DMP in 0.1 M sodium acetate buffer pH 4.5. All experiments were performed in triplicate. One unit of enzyme activity (U) was defined as the amount of enzyme transforming 1 µmol of substrate 2,6-DMP into 3,3’,5,5’-tetramethoxy-*p*-diphenoquinone (cerulignone) per minute under the given experimental conditions. The laccase activity was expressed as international units (U).

### Protein determination

Protein concentrations were determined by the method of Bradford^32^ with bovine serum albumin (Fluka) as standard.

### Oxidative biocatalysis

The laccase oxidation of benzyl alcohol to benzaldehyde was carried out at room temperature under magnetic stirring at 800 rpm in a final volume of 3 mL with 20 mM (0.06 mmol) benzyl alcohol, 6 mM TEMPO solution in 30 µL THF, 0.6 U laccase solution in 0.1 M citrate buffer at pH 5 with addition of oxygen. The addition to the reaction medium was performed at 0, 3, 6, and 24 h.

In preliminary experiments, the crude reaction mixture was analysed by H(1)-NMR, in which benzyl alcohol, benzaldeyde and benzoic acid were detected by three peculiar signals (the 8.16–8.13 multiplet for the benzoic acid; the 7.65 doublet for the benzaldehyde; the 4.64 broad singlet for the benzyl alcohol). Since no significant amount of benzoic acid was detected in these experiments, the benzaldehyde formation was measured with UV spectroscopy at 290 nm^33^ (Kawamura et al. 2016), a wavelength in which benzyl alcohol gave no absorption, in a 1-mL quartz cuvette against a calibration curve of the product between 0.2 and 10 mM. All experiments were performed in triplicate.

### Statistical analysis

Analysis of variance (ANOVA) of data from Benzaldehyde production was performed using Minitab ver.17 (Statistical software). Results were reported as mean of the production ± standard deviation (SD). Statistical differences (P < 0.05) among different laccases were determined according to Tukey’s test.

### Scale-up oxidation reaction of benzyl alcohol

10 mg of dialyzed liquor, in the lyophilized form, of *P. tigrinus 707* (corresponding to 14.2 U) were dissolved in 1 ml of citrate buffer 0.1 M pH 5. The solution was kept at room temperature for 1 h. In a 100 mL reaction flask a solution of benzyl alcohol (150 mg, 1.38 mmol), TEMPO (65 mg, 0.416 mmol, 30 mol%) in 0.69 ml of THF was mixed with 68,3 ml of 0.1 M citrate buffer at pH 5. 1 ml of laccase solution was added to this mixture. At this stage, the flask was charged with oxygen for about 10 min and was stirred at rt for 36 h. The crude reaction mixture was extracted with diethyl ether when conversion of benzyl alcohol is complete (TLC analysis). The combined organic layers were dried over anhydrous Na_2_SO_4_ and concentrated under vacuum. The crude reaction mixture was purified on a silica-gel chromatography to give 128 mg of benzaldehyde (Isolated Yield 85%).

### Ethical approval and consent to participate

This article does not contain any studies with human participants or animals performed by any of the authors.

## Results and discussion

### Production and purification of laccases

Four different white-rot fungi strains were grown on the agricultural residues composed of Saragolla straw:bran with a ratio 4:1 *w/w*. The collected laccase-containing liquor was further purified through different steps performing dialysis, ion exchange chromatography and gel filtration.

The maximum laccase production was obtained for all strains at the end of 7th day of fermentation with a specific activity of liquor of 6.4 U/mg protein for *Trametes versicolor* 11269, 14.5 U/mg for *Pleurotus ostreatus* 1020, 20.7 U/mg for *Panus tigrinus* 707 and 11.6 U/mg for *Lentinula edodes* SC-495. The crude laccase of the liquor was precipitated with 70% ammonium sulfate saturation, dialyzed, purified on ion-exchange chromatography and the pooled fractions further purified by gel filtration. A typical trend of increase of specific activity and purification fold was observed in all fungal laccases. Indeed, laccase from *Trametes versicolor* 11269, *Pleurotus ostreatus* 1020 and *Panus tigrinus* 707 exhibited a similar specific activity of 379.7 U/mg, 377.0 U/mg and 375.8 U/mg using 2,6-DMP at the standard assay conditions with an overall fold purification of 59.5, 26.0 and 18.1 and a yield of 17%, 30% and 16% respectively. Laccase from *Lentinula edodes* SC-495 was purified with the lowest specific activity of 118.2 U/mg with a 10.1 fold purification and a yield of 13% (Table 1).

**Table 1:** Purification profiles of laccases from different white rot fungi.

	Purification step	Volume (ml)	Total protein (mg)	Total activity (U)	Specific activity (U/mg)	Purification fold	Yield (%)
*Trametes versicolor* 11269	Liquor	150	283.4	1809.0	6.4	1.0	100
	Dialysis	30	136.8	1583.1	11.6	1.8	88
	Ion exchange	8	3.8	800.0	211.8	33.2	44
	Gel filtration	5	0.8	314.6	379.7	59.5	17
*Pleurotus ostreatus* 1020	Liquor	150	125.3	1813.5	14.5	1.0	100
	Dialysis	30	103.9	1258.5	12.1	0.8	69
	Ion exchange	8	7.8	769.8	98.8	6.8	42
	Gel filtration	10	1.5	552.2	377.0	26.0	30
*Panus tigrinus* 707	Liquor	150	167.0	3465.0	20.7	1.0	100
	Dialysis	30	128.7	3420.4	26.7	1.3	99
	Ion exchange	8	6.9	1121.3	162.5	7.8	32
	Gel filtration	10	1.5	552.4	375.8	18.1	16
*Lentinula edodes* SC-495	Liquor	150	197.0	2295.0	11.6	1.0	100
	Dialysis	30	137.2	1137.6	8.3	0.7	50
	Ion exchange	8	9.2	367.1	40.1	3.4	16
	Gel filtration	10	2.5	295.4	118.2	10.1	13

In this work, we compared the raw and purified laccases produced from mycelia, which were grown on agricultural residues, with commercially available enzyme in the oxidation of benzyl alcohol. We chose this reaction as a model of Laccase-catalyzed oxidation using TEMPO as a mediator. Since TEMPO has a poor solubility in water, a certain amount of organic co-solvent was needed. An excellent solvent for both protic and aprotic polar organic molecules is THF. THF is miscible in water, prevents the formation of undesired emulsions and has a limited volatility, if compared to acetone or ethyl acetate, a fact that is relevant when the reaction must last several days. Before testing our raw enzymes on the model reaction, it was necessary to evaluate their activity (tolerance) in the presence of different amounts of THF.

### Tolerance of laccase systems versus tetrahydrofuran (THF)

Dialyzed laccase solutions and commercial *Trametes versicolor* laccase were tested under different concentrations of THF to identify their specific tolerance level. Recently, it was reported that commercial laccases were employed also in water-miscible organic solvents ^34^.

**Figure 2:**
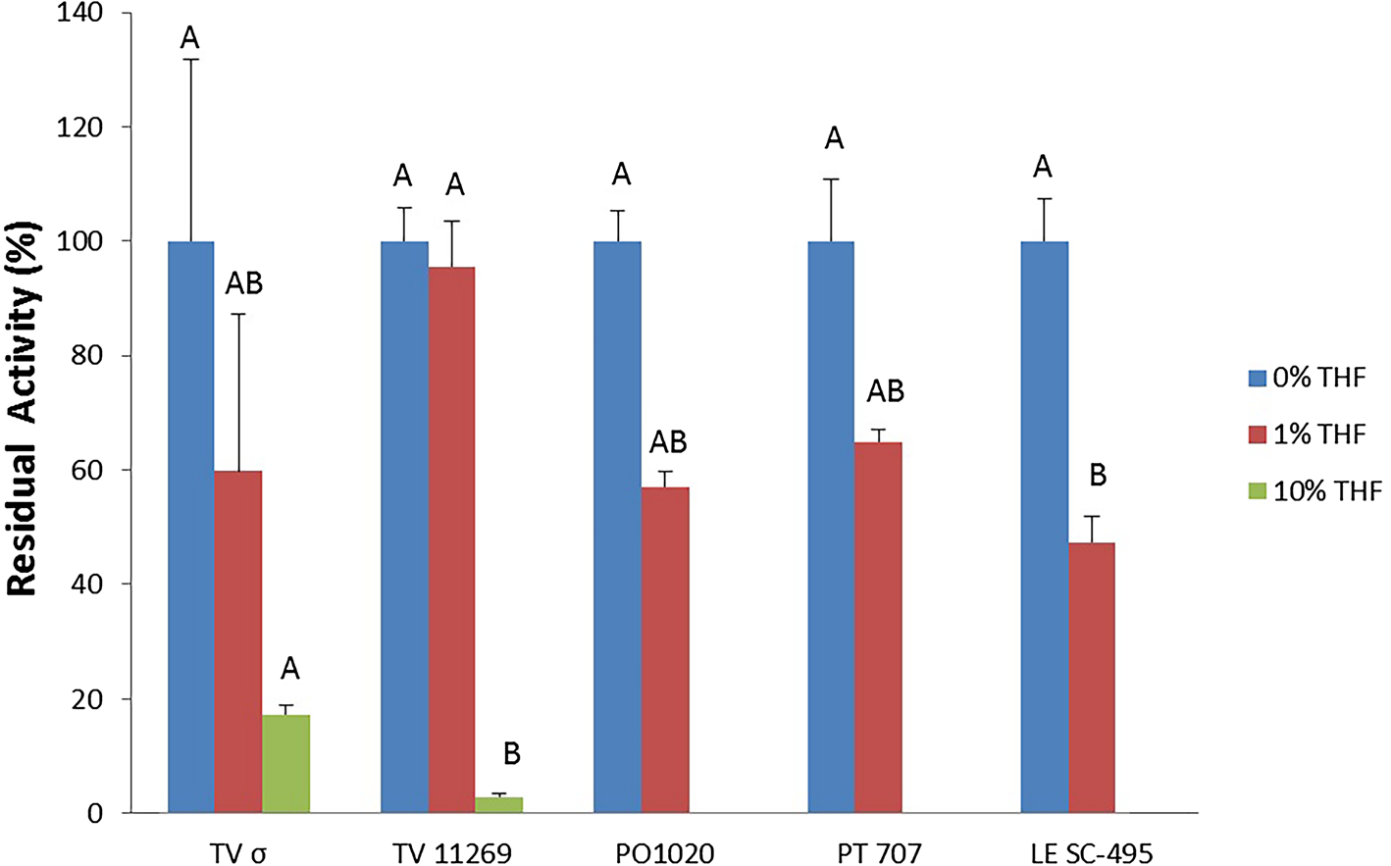
Tolerance to THF of the commercial laccase (TV σ) and the dialysate protein mixtures of TV 11269, PO 1020, PT 707, and LE SC-495. THF concentrations of 1% *v/v* and 10% *v/v* were tested and related to the laccase activity in aqueous solution. Data are mean values of three different measurements, different letters indicate differences according to Tukey Test at p < 0.05.

The commercial TV laccase (TV σ) and the dialysate (raw) protein mixtures of the four strains were assayed for their tolerance to THF at concentrations of 1% and 10% *v/v*, after 24 h incubation time and compared with the control without THF. The TV 11269 raw laccase solution showed the highest tolerance to the THF 1% *v/v*, which showed a 96% activity. The enzymatic activity is almost completely lost (3%) at a concentration of THF of 10% *v/v*. The commercial TV laccase showed a lower tolerance (59%) in THF at 1% *v/v* and a slightly higher tolerance at 10% THF (17%), compared to TV 11269 laccase. LE SC-495, PO 1020 and PT 707 laccase solutions were more affected by the presence of the THF co-solvent compared to the TV 11269 laccase solutions (Fig. 2). In fact, they showed lower residual activity with 47%, 57% and 65% residual activity respectively in 1% THF and not detectable activity at 10% THF (Fig. 2 and Table S1).

For this reason, the optimized 1% concentration of THF was used as the co-solvent for the biocatalytic model oxidation.

### Biocatalytic oxidation of benzyl alcohol

**Figure 3:**
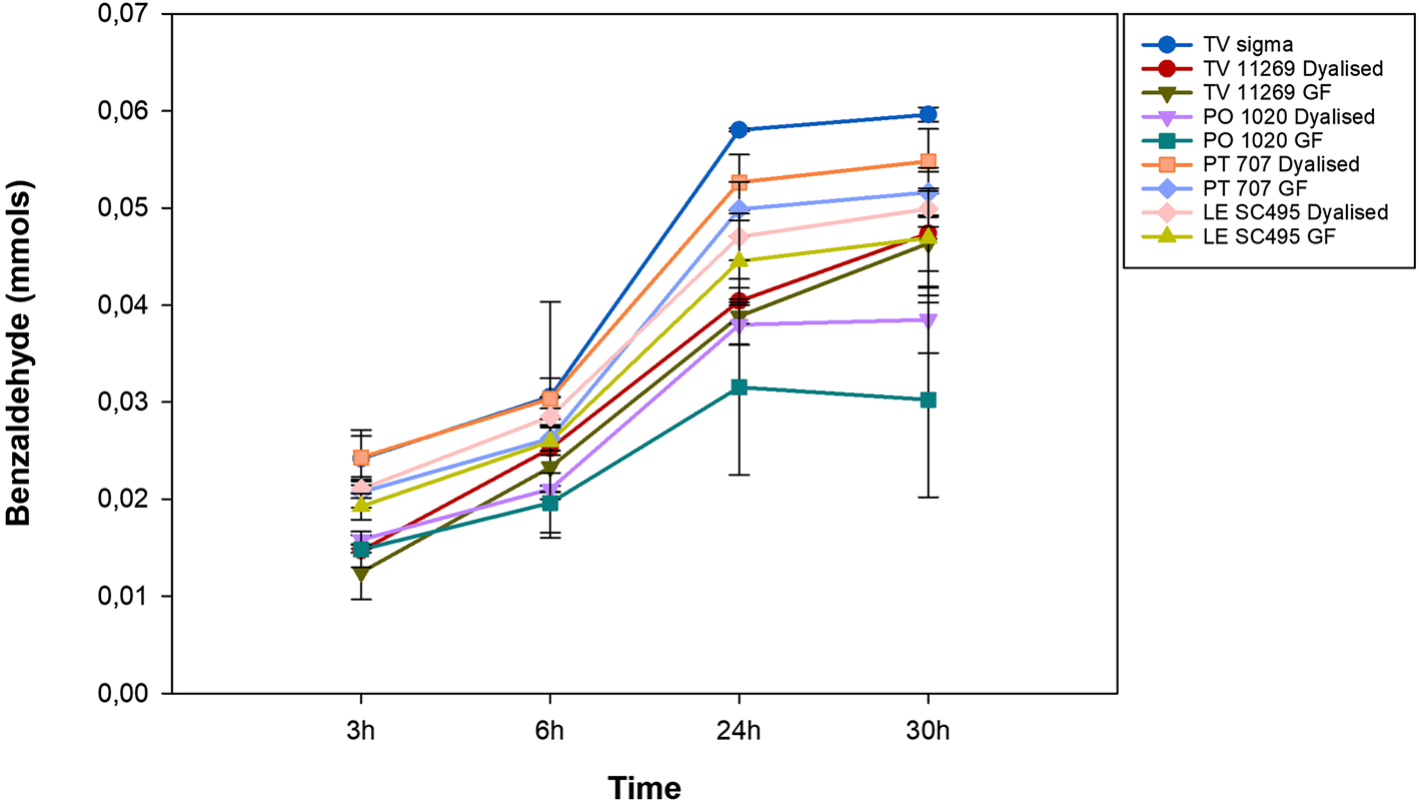
Time course of conversion of benzyl alcohol to benzaldehyde by dialyzed and gel filtrated laccase solutions produced from four different white-rot fungi compared to the commercial *T. versicolor* laccase (TV σ), after 3 h, 6 h, 24 h and 30 h from inoculation. Each point of time course represents mean of three different measurements with standard deviation.

The oxidation of benzyl alcohol to benzaldehyde was investigated as a model of a green chemistry process and of circular economy with possible future applications on an industrial scale. THF was used as the co-solvent with a minimal concentration of 1% (99:1 *v/v*) in the presence of 0.1 M sodium citrate buffer pH 5 at room temperature, with 0.06 mmol of benzyl alcohol. Oxygen was introduced throughout the entire incubation at different times. Aliquots of the dialysis and the gel filtration products were used on the reaction. The amount of benzaldehyde obtained by oxidation of benzyl alcohol through laccases was measured after 3 h, 6 h, 24 h and 30 h (Fig. 3, Table S2).

The data indicate that the benzylic alcohol oxidation rates are not constant over time, and, in most cases, the enzyme appears to be more active as time progresses until 24 h, when the reaction reaches the plateau. Only in the case of dialyzed and GF (Gel filtrated) TV11269 laccases we observed a linearity in the benzaldehyde production and the curve seems to continue growing after 24 h.

**Figure 4:**
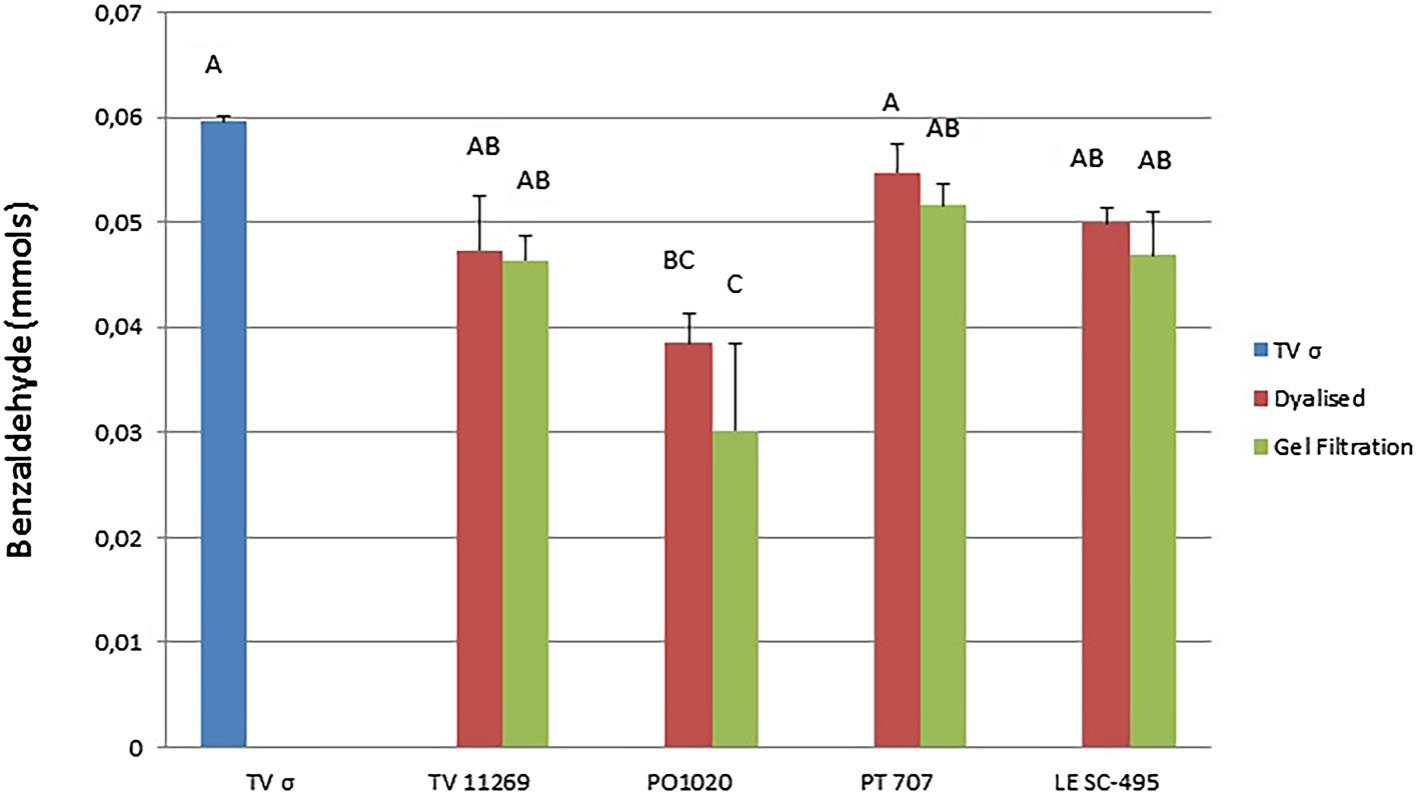
Conversion of benzyl alcohol to benzaldehyde by laccase solutions produced from four different white-rot fungi compared to the commercial *T. versicolor* laccase (TV σ), after 30 h from inoculation. Data are mean values of three different measurements, different letters indicate differences according to Tukey test at p < 0.05.

The highest performance was observed for the commercial *T. versicolor* laccase (TV σ) with a 99% of conversion in product after 30 h. Nevertheless, no significant differences were shown about the activity of laccases from all the tested strains and for both steps of purification with exception of *P. ostreatus* 1020 (Fig. 4 and Tables S2 and S3).

PT 707 laccase showed 91% of conversion for the dialyzed laccase against 86% of conversion produced by the gel filtered enzyme, followed by LE SC 495 with an 83% of conversion for the dialyzed laccase against 78% of conversion of the gel filtration product and TV 11269 that achieved a 79% of conversion for the dialyzed laccase against 77% of conversion of the gel filtration product. The lowest conversion was recorded by the laccase solutions from PO 1020 which showed a 64% of conversion for the dialyzed laccase against 50% of conversion of the gel filtration product.

Those data show that purification steps are not justified by a substantial activity increase of enzyme solutions in the biocatalytic transformation of benzyl alcohol. Thus, raw liquor squeezed from fungal fermented wheat straw can be considered a useful biocatalyst reducing the production times and costs for possible future use of these in industrially relevant biocatalysed process.

## Conclusions

We have demonstrated that solid-state fermentation (SSF) is a successful technique to cultivate white-rot fungi on agricultural residues to produce laccase-containing solutions which are tolerant to THF at a concentration of 1% (99:1 *v/v*)*.* The activity of crude and purified laccase-containing mixtures produced from four edible mushrooms which were grown using wheat straw as raw material was compared on the model reaction of the green oxidation of benzyl alcohol to benzaldehyde through the laccase/TEMPO (LMS) in H_2_O-1%THF. Biomass and agro-industrial waste, indeed, can be a great alternative to produce chemicals and biocatalysts which can increase the sustainability of industrial processes. Further studies will be performed to characterize the composition of the dialysate and to extend the process to the sustainable and green production of industrially relevant organic building-blocks.

## Supplementary Information

Supplementary Information.

## Data Availability

All data generated during the current study are included into this published paper.
