# Supplementary material for: A comparative screening of laccase-mediator systems by white-rot fungi laccases for biocatalytic benzyl alcohol oxidation
Source: Sci Rep. 2022 Dec 14;12:21602. doi: 10.1038/s41598-022-24839-6 (PMC9751274; doi:10.1038/s41598-022-24839-6)
Supplement: Supplementary file 1 — Supplementary Information. [file 41598_2022_24839_MOESM1_ESM.pdf]

A comparative screening of laccase-mediator systems by white-rot fungi laccases for biocatalytic alcohol oxidation

Ivana Marino[1*], Eugenia Pignataro[1,2†], Donatella Danzi[5], Francesco Cellini[1], Cosimo Cardellicchio[3], Antonino Biundo[4], Isabella Pisano[4], Maria Annunziata M. Capozzi[2*]

[1] Agenzia Lucana di Sviluppo e Innovazione in Agricoltura, Centro Ricerche Metapontum Agrobios, SS. Jonica 106, Km 448,2 75012 Bernalda, Italy

[2] Department of Chemistry, University of Bari Aldo Moro, Via Edoardo Orabona 4, 70125 Bari, Italy

[3] CNR ICCOM, Department of Chemistry, University of Bari Aldo Moro, Via Edoardo Orabona 4, 70125 Bari, Italy

[4] Department of Biosciences, Biotechnology and Biopharmaceutics, University of Bari Aldo Moro, Via Edoardo Orabona 4, 70125 Bari, Italy

[5] Institute of Biosciences and Bioresources, National Research Council, 70126 Bari, Italy

[*] Corresponding author(s): Email(s): maria.capozzi@uniba.it; ivana.marino@alsia.it

Supporting information

Figure S1. SDS-PAGE analysis of different purification fractions of TV 11269 laccase. Pag. 2

Table S1: ONE WAY ANOVA analysis for the tolerance to THF of laccases Pag. 3

Table S2: Benzyl alcohol oxidation to benzaldehyde over time using laccase - TEMPO Pag. 3

Table S3: ONE WAY ANOVA analysis for Benzaldehyde production after 30h of reaction Pag. 5

Table S4– Coefficient of Correlation (R) and Determination (R^2) Pag. 6

References Pag.6

SDS-PAGE analysis of different purification fractions of TV 11269 laccase

The SDS PAGE of the different steps of Trametes versicolor 11269 laccase purification, confirms that there is an increase in the purity of the laccase solution, as shown also in Figure. Although with some impurities, one band at around 40 kDa and two bands around 100 KDa in different purification steps and in different fractions. The results suggested that the enzyme is a monomeric protein with a molecular mass of ca. 60KDa, as previously reported (Youshuang et al. 2011). Fractions with the same purity grade were pooled to increase the amount of laccase for bio catalytic oxidation of benzyl alcohol.

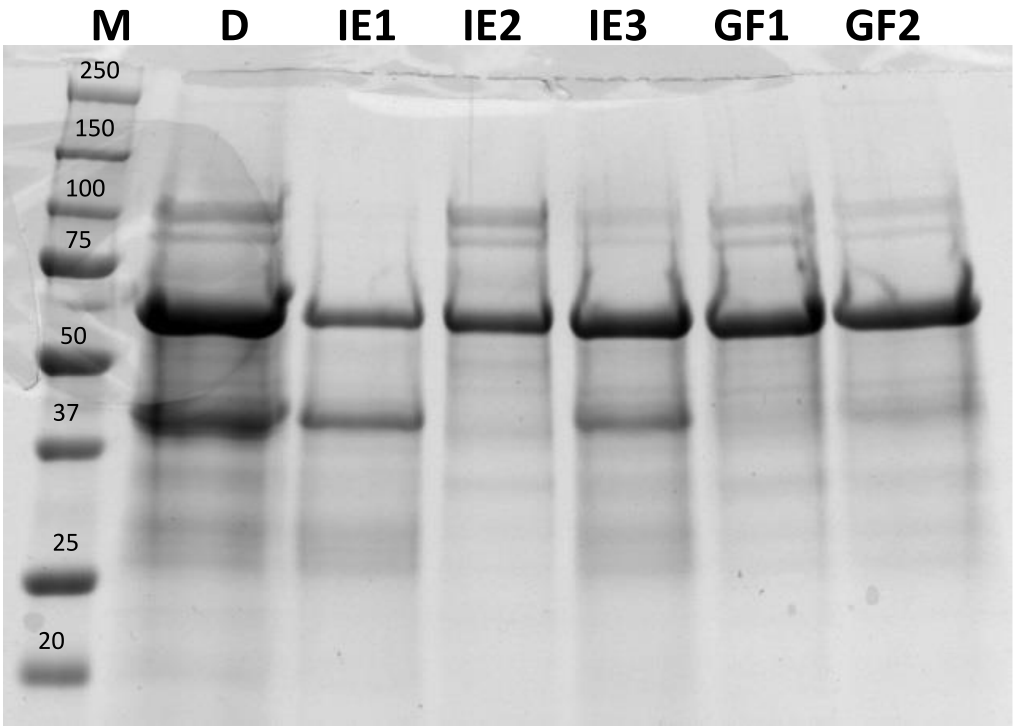

Figure S1 SDS-PAGE analysis of different purification fractions of TV 11269 laccase. Lane M, Precision Plus Protein™ All Blue (Bio-Rad, USA); lane D, Dialysis fraction; lane IE1, IE2, and IE3, Ion-exchange fractions; Lane GF1 and GF2, Gel-Filtration fractions. Image acquired with ChemiDoc^{TM} Touch Imaging System (BioRad)

ONE WAY ANOVA analysis for the tolerance to THF of the different laccases

	0% THF	1% THF	10% THF
**TV σ**	100.00^{A}(±31.9)	59.8^{AB}(±27.5)	17.32^{A}(±1.50)
**TV 11269**	100.00^{A}(±5.80)	95.56^{A}(±7.96)	2.90^{B}(±0.58)
**PO 1020**	100.00^{A} (±5.31)	57.02^{AB} (±2.66)	-
**PT 707**	100.00^{A} (±10.81)	64.95^{AB} (±2.09)	-
**LE SC495**	100.00^{A}(±7.55)	47.35^{B}(±4.66)	-
**ONE WAY ANOVA**	F=0.03; *p*=0.999	F=4.25; *p*=0.029	F=90.79; *p*=0.002

Table S1: Mean and ONE WAY ANOVA analysis for the tolerance to THF of the commercial laccase (TV σ) and the dialysate protein mixtures of TV 11269, PO 1020, PT 707, and LE SC-495. F statistic represents the ratio between estimated population variance between groups and estimated population variance within groups. Different letters indicate differences according to Tukey test at p<0,05. Standard deviations are reported in brackets.

Benzyl alcohol oxidation to benzaldehyde over time using laccase - TEMPO as catalyst

	**Benzaldehyde [mmol]**			
	**3h**	**6h**	**24h**	**30h**
	0,02149823737	0,01929452278	0,05789629847	0,059868543
	0,02531433608	0,03535618227	0,05804755843	0,058794765
	0,02571647735	0,03701201201	0,05819517887	0,060213451
**TV sigma**	0,02417635026	0,03055423902	0,05804634526	0,05962558633
	0,01451919311	0,01923299386	0,04136016451	0,05390080298
	0,01460830396	0,02759524742	0,03780185076	0,04121771772
	0,01493341167	0,02885233059	0,04205787309	0,04702348544

**TV 11269 Dyalised**	0,01468696958	0,02522685729	0,04040662946	0,04738066871
	0,01045926361	0,01502630892	0,03550406385	0,04968388497
	0,011274971	0,026183	0,0400438	0,045000473
	0,01568644732	0,02863461287	0,04098340188	0,04442312965
**TV 11269 GF**	0,01247356064	0,02328130726	0,03884375524	0,04636916254
	0,01621034078	0,02143288941	0,03956937916	0,04110686774
	0,01597434391	0,02077784306	0,03865305523	0,03976743047
	0,01528593811	0,02086493015	0,03569942225	0,03459443139
**PO 1020 Dyalised**	0,01582354093	0,02102522087	0,03797395221	0,03848957653
	0,01276145711	0,01616033425	0,02197316882	0,02000380272
	0,01540834313	0,02199706228	0,03998800431	0,04006303042
	0,01628084606	0,02068696958	0,03268532119	0,03065925708
**PO 1020 GF**	0,0148168821	0,0196147887	0,03154883144	0,03024203007
	0,02102624363	0,02842068155	0,05231418919	0,05448276537
	0,02621621622	0,03258003656	0,05566319036	0,05828399595
	0,02563258911	0,03006965661	0,0499559342	0,05165609087
**PT 707 Dyalised**	0,02429168299	0,03035679157	0,05264443792	0,0548076174
	0,0200097924	0,02604850503	0,05311887975	0,0534804968
	0,01961222092	0,02508676067	0,04835120447	0,05266759694
	0,02253035644	0,02759714062	0,04817910302	0,04871856639
**PT 707 GF**	0,02071745659	0,02624413544	0,04988306241	0,05162222005
	0,02028887583	0,02755170388	0,04476963376	0,04778096031
	0,02212593028	0,02910223267	0,04673717195	0,05092170975
	0,02071680376	0,02884097141	0,049560566	0,05105565348
**LE SC495 Dyalised**	0,02104386996	0,02849830265	0,04702245724	0,04991944118
	0,0176831179	0,0242954041	0,0428858206	0,04433537342

	0,02001762632	0,02657292075	0,0493140423	0,05280615942
	0,02004700353	0,02694777386	0,04135086173	0,0435732798
**LE SC495 GF**	0,01924924925	0,02593869957	0,04451690821	0,04690493755

Table S2: Benzyl alcohol oxidation to benzaldehyde over time using laccase - TEMPO as catalyst.
Initial quantity of benzyl alcohol: 0.06 mmol. Aliquots of 10 µL were taken periodically to quantify spectrophotometrically the amount of benzaldehyde. For every fungal strain, reaction was carried out using laccase in the dyalised product and in the gel filtration (GF), in triplicate.

ONE WAY ANOVA analysis for the mmols Benzaldehyde production after 3, 6, 12, 30 h from inoculum

	Mean			
	**3h**	**6h**	**12h**	**30h**
**LE SC495 Dyalised**	0,02104^{AB}(±0,000961)	0,02849^{A} (±0,000830)	0,04702^{ABCD} (±0,00241)	0,04992^{AB}(±0,001513)
**LE SC495 GF**	0,01925^{ABC}(±0,001356)	0,02594^{A} (±0,001435)	0,04452^{BCD} (±0,00422)	0,04690^{AB}(±0,004184)
**PO 1020 Dyalised**	0,01583^{BCD}(±0,000480)	0,02103^{A}(±0,000356)	0,03797^{DE}(±0,00202)	0,03849^{BC}(±0,002808)
**PO 1020 GF**	0,01482^{CD} (±0,00183)	0,01961^{A}(±0,00306)	0,03155^{E}(±0,00906)	0,03024^{C} (±0,008195)
**PT 707 Dyalised**	0,02429^{A} (±0,00284)	0,03036^{A}(±0,00209)	0,05264^{AB}(±0,00287)	0,05481^{A} (±0,002716)
**PT 707 GF**	0,02072^{AB}(±0,001583)	0,02624^{A}±0,001267)	0,04988^{ABC}(±0,00280)	0,05162^{AB}(±0,002080)
**TV 11269 Dyalised**	0,01469^{CD}(±0,000218)	0,02523^{A}(±0,00523)	0,04041^{CDE}(±0,00228)	0,04738^{AB}(±0,005184)
**TV 11269 GF**	0,01247^{D}(±0,00281)	0,02328^{A}(±0,00725)	0,03884^{CDE}(±0,00293)	0,04637^{AB}(±0,002356)
**TV sigma**	0,02418^{A} (±0,00233)	0,03055^{A} (±0,00979)	0,05805^{A} (±0,000149)	0,05963^{A} (±0,000604)
**ONE WAY ANOVA**	F=16,61; *p*<0,001	F=2,06; *p*=0,097	F=13,14; *p*<0,001	F=9,75; *p*<0,001

Table S3: Mean and ONE WAY ANOVA analysis for the mmols Benzaldehyde production after 3, 6, 12 and 30 h from inoculum.
F statistic represents the ratio between estimated population variance between groups and estimated population variance within groups. Different letters indicate differences according to Tukey test at p<0,05. Standard deviations are reported in brackets.

Coefficient of Correlation (R) and Determination (R^2)

-	R	R^2
**TV sigma**	0,9876	0,9753
**TV 11269 Dyalised**	0,9792	0,9589
**TV 11269 GF**	0,9796	0,9595
**PO 1020 Dyalised**	0,9821	0,9645
**PO 1020 GF**	0,9578	0,9173
**PT 707 Dyalised**	0,9896	0,9792
**PT 707 GF**	0,9889	0,978
**LE SC495 Dyalised**	0,9872	0,9746
**LE SC495 GF**	0,9878	0,9758

Table S4: Coefficient of Correlation (R) and Determination (R^2).
Coefficient correlation represents the degree of relationship between production of Benzaldehyde and time. The square of Coefficient of Correlation is the Coefficient of Determination. R^2 shows percentage variation in y (benzaldehyde production) which is explained by all the y variables. It can go between 0 and 1. The higher coefficients are shown for PT 707 Dyalised, and the lower for PO 1020 GF. Overall a high linear relation is shown for all laccases.

References

Youshuang Z, Haibo Z, Mingle C, Zhenzhen W, Feng H, Peiji G (2011) Production of a thermostable metal-tolerant laccase from Trametes versicolor and its application in dye decolorization. Biotechnol Bioproc E 16, 1027-1035. https://doi.org/10.1007/s12257-011-0129-0
